# Pneumococci remain the main cause of complicated pediatric pneumonia in the post-pandemic era despite extensive pneumococcal vaccine use

**DOI:** 10.1186/s41479-024-00151-x

**Published:** 2024-11-25

**Authors:** Joana Gomes-Silva, Marcos D. Pinho, Ana Friães, Mário Ramirez, José Melo-Cristino, Catarina Silva-Costa, the Portuguese Group for the Study of Streptococcal Infections, Margarida Pinto, Miguel Seruca, João Marques, Isabel Peres, Teresa Pina, Isabel Lourenço, Cristina Marcelo, Isabel Daniel, Odete Chantre, Vasco Mendes, Marília Gião, Rui Ferreira, Rui Tomé Ribeiro, Celeste Pontes, Luísa Boaventura, Teresa Reis, Henrique Oliveira, Catarina Chaves, Mariana Silva, Ana Aguiar, Hugo Loureiro, Adriana Pedrosa, Hermínia Costa, Maria Fátima Silva, Maria Amélia Afonso, Mariana Fardilha, Natália Novais, Isabel Brito, Luís Marques Lito, Ana Bruschy Fonseca, Maria Ana Pessanha, Elsa Gonçalves, Teresa Morais, Cristina Toscano, Elisabete Cristovam, Paulo Lopes, Angelina Lameirão, Gabriela Abreu, Aurélia Selaru, Ana Paula Mota Vieira, Margarida Tomaz, Cláudia Ferreira, Marta Nicolau, Ana Paula Castro, Virgínia Lopes, Hugo Cruz, Fernando Fonseca, Nádia Martins, Carla Leite, Ana Paula Castro, Filipa Vicente, Margarida Pereira, Ilse Fontes, Maria Paula Falcão, Rui Semedo, Gina Marrão, Filipa Silva, Manuela Ribeiro, Helena Gonçalves, Alberta Faustino, Maria Cármen Iglesias, Adriana Coutinho, Ana Bela Correia, Luísa Gonçalves, Elzara Aliyeva, Sandra Schäfer, Clara Portugal, Isabel Monge, José Diogo, Filipa Fortunato, Leonardo Carneiro, José Marta, Nadiya Kruptsala, Cláudia Fidalgo, Raquel Diaz, Sónia Ferreira, Inês Cravo Roxo, Isabel Vale, Maria João Tomás, Maria Antónia Read, Valquíria Alves, Margarida Monteiro, João Faria, José Mota Freitas, Sandra Vieira, Elsa Calado, Bruno Miguel, L Nogueira Martins, Maria Favila Menezes, Maria José Rego de Sousa, Maria Calle, Mariana Bettencourt Viana, Marvin Oliveira, Hugo Macedo, Vitória Rodrigues, Sofia Marques, Joana Selada, Patrícia Pereira, Manuela Azevedo, Jesuína Duarte, Joana Bernardo, Inês Tapadinhas, Ana Filipa Resende, Andreia Bernardo, Luísa Oliveira, Susana Banza, Ezequiel Moreira, Carla Ferreira, Adília Vicente, Cristina Bragança, Maria Lucas, Paula Gouveia Pestana, Patrícia Amantegui, Cristina Mota Preto, Sara F. Sampaio, Ana Jesus, Marisol Lourinha, the Portuguese Study Group of Invasive Pneumococcal Disease of the Pediatric Infectious Disease Society, Catarina Gouveia, Teresa Tomé, Mónica Rebelo, Ana Teixeira, Maria João Virtuoso, Nancy Guerreiro, Fernanda Rodrigues, Cristina Resende, Sónia Aires, Agostinho Fenandes, Filipa Prata, Marisa Vieira, Rita Morais, Diana Moreira, Isabel Carvalho, Alexandra Costa, Ana Teixeira, Cristina Ferreira, Graça Seves, Laura Marques, Ana Braga, Margarida Guedes, Maria José Dinis, Eurico Gaspar, Bernardo Camacho, Céu Novais, Maria Manuel Zarcos, Margarida Tavares, Manuela Costa Alves, Sofia Lima, Carla Cruz, Manuela Brandão, Paula Correia, Sofia Fraga, João Franco, Sílvia Almeida, Cristina Faria, Sofia Arosa, Florbela Cunha, Hugo Rodrigues, Joaquim Cunha, Cláudia Monteiro, Estela Veiga, Fernanda Pereira, Manuela Ferreira, Álvaro Sousa, Francisca Lopes, Sara Santos, Ana Luísa Teixeira, Fernanda Marcelo, Pedro Carvalho, Filomena Pereira, Gustavo Rodrigues, Marta Cabral, Maria Ana S. Nunes, Pedro Flores, Manuel Cunha, Dora Gomes, João Calado Nunes, Rosário Massa, Fátima Nunes, Isabel Monteiro, Cristina Didelet, António Salgado, Luís Gonçalves

**Affiliations:** 1 Instituto de Medicina Molecular, Faculdade de Medicina, Universidade de Lisboa, Av. Prof. Egas Moniz, Lisbon, PT 1649-028 Portugal; 2 Unidade Local de Saúde de São José, Lisboa, Portugal; 3 Unidade Local de Saúde Algarve, Faro e Portimão, Portugal; 4 Unidade Local de Saúde Coimbra, Coimbra, Portugal; 5 Unidade Local de Saúde Entre Douro e Vouga, Sta Maria da Feira, Portugal; 6 Unidade Local de Saúde Baixo Mondego, Figueira da Foz, Portugal; 7 Unidade Local de Saúde Santa Maria, Lisboa, Portugal; 8 Unidade Local de Saúde Lisboa Ocidental, Lisboa, Portugal; 9 Unidade Local de Saúde Gaia/Espinho, Vila Nova de Gaia e Espinho, Portugal; 10 Unidade Local de Saúde Alto Ave, Guimarães, Portugal; 11 Unidade Local de Saúde Baixo Alentejo, Beja, Portugal; 12 Unidade Local de Saúde Santo António, Porto, Portugal; 13 Unidade Local de Saúde Póvoa do Varzim/Vila do Conde, Póvoa do Varzim e Vila do Conde, Portugal; 14 Unidade Local de Saúde Trás-os-Montes e Alto Douro, Vila Real, Peso da Régua e Chaves, Portugal; 15 Serviço de Saúde da Região Autónoma da Madeira, Funchal, Portugal; 16 Unidade Local de Saúde Alto Alentejo, Elvas e Portalegre, Portugal; 17 Unidade Local de Saúde Região de Leiria, Leiria, Portugal; 18 Unidade Local de Saúde de São João, Porto, Portugal; 19 Unidade Local de Saúde Braga, Braga, Portugal; 20 Unidade Local de Saúde Alentejo Central, Évora, Portugal; 21 Hospital dos SAMS, Lisboa, Portugal; 22 Unidade Local de Saúde Amadora/Sintra, Amadora e Sintra, Portugal; 23 Unidade Local de Saúde Almada/Seixal, Almada e Seixal, Portugal; 24 Unidade Local de Saúde Região de Aveiro, Aveiro, Portugal; 25 Unidade Local de Saúde Viseu, Dão - Lafões, Tondela e Viseu, Portugal; 26 Unidade Local de Saúde Matosinhos, Matosinhos, Portugal; 27 Unidade Local de Saúde Estuário do Tejo, Vila Franca de Xira, Portugal; 28 Unidade Local de Saúde Alto Minho, Ponte de Lima e Viana do Castelo, Portugal; 29 Centro de Medicina Laboratorial Germano de Sousa, Lisboa, Portugal; 30 Unidade Local de Saúde Tâmega e Sousa, Amarante e Guilhufe, Portugal; 31 Laboratório Synlab, Lisboa, Portugal; 32 Unidade Local de Saúde Arrábida, Setúbal, Portugal; 33 Unidade Local de Saúde Médio Ave, Santo Tirso e Vila Nova de Famalicão, Portugal; 34 Unidade Local de Saúde Oeste, Caldas da Rainha, Portugal; 35 Unidade Local de Saúde Cova da Beira, Covilhã, Portugal; 36 Centro de Medicina Laboratorial Germano de Sousa Açores, Ponta Delgada, Portugal; 37 Unidade Local de Saúde do Arco Ribeirinho, Barreiro e Montijo, Portugal; 38 Hospital Particular do Algarve, Faro, Portugal; 39 Unidade Local de Saúde Loures/Odivelas, Loures, Portugal; 40 Unidade Local de Saúde do Nordeste, Bragança, Portugal; 41 Unidade Local de Saúde Castelo Branco, Castelo Branco, Portugal; 42 Unidade Local de Saúde Guarda, Guarda, Portugal; 43 Instituto Português de Oncologia, Lisboa, Portugal; 44 Hospital Lusíadas, Lisboa, Portugal; 45 Hospital da Luz, Lisboa, Portugal; 46 Hospital da Cruz Vermelha, Lisboa, Portugal; 47 Hospital CUF Descobertas, Lisboa, Portugal; 48 Hospital de Cascais, Cascais, Portugal; 49 Hospital da Horta, Horta, Portugal; 50 Unidade Local de Saúde da Lezíria, Santarém, Portugal; 51 Unidade Local de Saúde do Médio Tejo, Abrantes, Tomar e Torres Novas, Portugal; 52 Hospital do Santo Espírito, Angra do Heroísmo, Portugal; 53 Hospital do Divino Espírito Santo, Ponta Delgada, Portugal

**Keywords:** Streptococcus pneumonia, Empyema, Vaccines, Molecular Diagnostics, Serotypes, Epidemiology, Pediatric infectious disease

## Abstract

Nucleic acid amplification tests (NAATs) greatly enhance the capacity to identify the etiology of pediatric complicated pneumonia. However, the use of pneumococcal conjugate vaccines could reduce the importance of *Streptococcus pneumoniae* in pediatric complicated pneumonia with the potential emergence of other bacterial agents. Using an expanded NAAT in culture negative pleural fluid or empyema samples collected in 2010–2024 (*n* = 554) in Portugal, we show that *S. pneumoniae* remains the most frequent agent despite decades of pneumococcal conjugate vaccine use and the COVID-19 pandemic. A rebound in pediatric complicated pneumonia occurred post-pandemic, including a rise in cases by *Streptococcus pyogenes* and *Haemophilus influenzae*. Empiric therapy of pediatric complicated pneumonia should still consider *S. pneumoniae* as the most likely cause, even in countries where the pneumococcal conjugate vaccine is in the national immunization program with a high uptake.

**Supplementary Information:**

The online version contains supplementary material available at 10.1186/s41479-024-00151-x.

## Introduction

The advent of pneumococcal conjugate vaccines (PCVs), although including only a fraction of the > 100 serotypes known, raised the possibility of effectively preventing a significant part of pneumonias, including complicated cases. The initial 7-valent PCV was mostly targeting serotypes identified in blood, irrespective of disease presentation, but the 13-valent PCV (PCV13) already included the most frequent serotypes identified in pediatric complicated pneumonias (pneumonias occurring with parapneumonic effusion or empyema, PCP).

The etiologic diagnosis of PCP remains challenging, with culture of empyema, pleural fluid or blood being frequently negative and highlighting the importance of nucleic acid amplification tests (NAATs) [1–3]. Several bacteria are reported frequently as causing PCP, including: *Streptococcus pneumoniae, Staphylococcus aureus* (methicillin resistant and susceptible), *Streptococcus pyogenes* (or Lancefield group A streptococcus – GAS), *Haemophilus influenzae*, *Mycoplasma pneumoniae*, *Mycobacterium tuberculosis* and other species of the *Streptococcus* genus [4, 5]. Notwithstanding the dominance of *S. pneumoniae* as a causative agent of PCP in Portugal, the number of samples testing negative for *S. pneumoniae* increased from 43/135 in 2010–2015 to 76/163 in 2016–2019 (*p* = 0.013) [1, 2], raising the possibility of increases of other bacterial species as causes of PCP. This, together with a high PCV13 uptake in Portugal (> 95%), led us to expand our NAAT to identify other possible bacterial causes of these infections.

## Methods

We included culture-negative empyema and pleural fluid samples from children and adolescents (pediatric patients) (< 18 years) recovered in 52 hospital laboratories and pediatric departments located throughout Portugal. This is a service offered by the central laboratory to participating hospitals and samples are collected prospectively.

For all the samples received, we performed four real-time PCR (rPCR) multiplex reactions targeting different bacterial species (Table 1) and simultaneously detecting human DNA [1].

**Table 1:** Primers and probes used in the nucleic acid amplification test

Organism	Target	Sequence		Reference
*Streptococcus pneumoniae*	*lytA*	Primer Forward	ACGCAATCTAGCAGATGAAGCA	Silva-Costa C, Gomes-Silva J, Pinho MD, Friães A, Ramirez M, Melo-Cristino J. Continued Vaccine Breakthrough Cases of Serotype 3 Complicated Pneumonia in Vaccinated Children, Portugal (2016–2019). Microbiol Spectr. 2022; e0107722. [1]
		Primer Reverse	TCGTGCGTTTTAATTCCAGCT	
		Probe	TGCCGAAAACGCTTGATACAGGGAG	
	wzg	Primer Forward	GCTGTTTTAGCAGATAGTGAGATCGA	Silva-Costa C, Gomes-Silva J, Pinho MD, Friães A, Ramirez M, Melo-Cristino J. Continued Vaccine Breakthrough Cases of Serotype 3 Complicated Pneumonia in Vaccinated Children, Portugal (2016–2019). Microbiol Spectr. 2022; e0107722. [1]
		Primer Reverse	TCCCAGTCGGTGCTGTCA	
		Probe	AATGTTACGCAACTGACGAG	
*Streptococcus pyogenes*	*spy*	Primer Forward	GCACTCGCTACTATTTCTTACCTCAA	Pernica JM, Moldovan I, Chan F, Slinger R. Real-Time Polymerase Chain Reaction for Microbiological Diagnosis of Parapneumonic Effusions in Canadian Children. Canadian Journal of Infectious Diseases and Medical Microbiology. 2014; 25: 151–154
		Primer Reverse	GTCACAATGTCTTGGAAACCAGTAAT	
		Probe	CCGCAACTCATCAAGGATT TCTGTTACCA	
*Staphylococcus aureus*	*nuc*	Primer Forward	AAATTACATAAAGAACCTGCGACA	Edin A, Granholm S, Koskiniemi S, Allard A, Sjöstedt A, Johansson A. Development and laboratory evaluation of a real-time PCR assay for detecting viruses and bacteria of relevance for community-acquired pneumonia. J Mol Diagn. 2015;17: 315–324
		Primer Reverse	GAATGTCATTGGTTGACCTTTGTA	
		Probe	AATTTAACCGTATCACCATCAATCGCTTT	
*Haemophilus influenzae*	*siaT*	Primer Forward	AATGCGTGATGCTGGTTATGAC	Price EP, Harris TM, Spargo J, Nosworthy E, Beissbarth J, Chang AB, et al. Simultaneous identification of *Haemophilus influenzae* and *Haemophilus haemolyticus* using real-time PCR. Future Microbiology. 2017; 12: 585–593
		Primer Reverse	AAGAGTTTTGCGATAGATTCATTGG	
		Probe	AGAAGCAGCAGTAATT	
*Mycoplasma pneumoniae*	P1	Primer Forward	GGAATCCCAATGCACAAGAACA	Edin A, Granholm S, Koskiniemi S, Allard A, Sjöstedt A, Johansson A. Development and laboratory evaluation of a real-time PCR assay for detecting viruses and bacteria of relevance for community-acquired pneumonia. J Mol Diagn. 2015; 17: 315–324
		Primer Reverse	GCTTTGGTCAACACATCAACCTT	
		Probe	AACTCTTACGCAATCTAGCAGATGAA	
*Mycobacterium tuberculosis*	*IS6110*	Primer Forward	GTCGAACGGCTGATGACCAAACT	Choi Y, Jeon B-Y, Shim TS, Jin H, Cho S–N, Lee H. Development of a highly sensitive one-tube nested real-time PCR for detecting *Mycobacterium tuberculosis*. Diagnostic Microbiology and Infectious Disease. 2014; 80: 299–303
		Primer Reverse	TCCGAAGCGGCGCTGGACGA	
		Probe	ACCACGATCGCTGATCCGGCCACA	
*Streptococcus agalactiae*	*cfb*	Primer Forward	GGGAACAGATTATGAAAAACCG	Diaz MH, Waller JL, Napoliello RA, Islam MS, Wolff BJ, Burken DJ, et al. Optimization of Multiple Pathogen Detection Using the TaqMan Array Card: Application for a Population-Based Study of Neonatal Infection. PLOS ONE. 2013; 8: e66183
		Primer Reverse	AAGGCTTCTACACGACTACCAA	
		Probe	AGACTTCATTGCGTGCCAACCCTGAGAC	
Human (positive control)	RNAseP	Primer Forward	CCAAGTGTGAGGGCTGAAAAG	Centers for Disease Control and Prevention (CDC). PCR for Detection and Characterization of Bacterial Meningitis Pathogens: *Neisseria meningitidis*, *Haemophilus influenzae*, and *Streptococcus pneumoniae*. Available: http://www.cdc.gov/meningitis/lab-manual/chpt10-pcr.pdf
		Primer Reverse	TGTTGTGGCTGATGAACTATAAAAGG	
		Probe	CCCCAGTCTCTGTCAGCACTCCCTTC	

## Results

Between July 2010 and June 2024, we analyzed 544 samples. Up to 2020, samples were tested prospectively solely for *S. pneumoniae* and the results were reported previously [1, 2]. These samples were in storage and were re-tested retrospectively with the reactions targeting the additional six pathogens. From 2021 onwards samples were tested prospectively. The age of the patients ranged from 2 months to 17 years (data missing for 10 patients). 197 samples were negative for all bacteria tested (36.2%). In eight samples, we were not able to perform all PCR reactions due to limited sample availability, with the majority being positive for *S. pneumoniae* (6/8). The number of samples analyzed each year and positive identifications in the NAAT are presented in additional Table 1 [see Additional file 1].

**Fig. 1:**
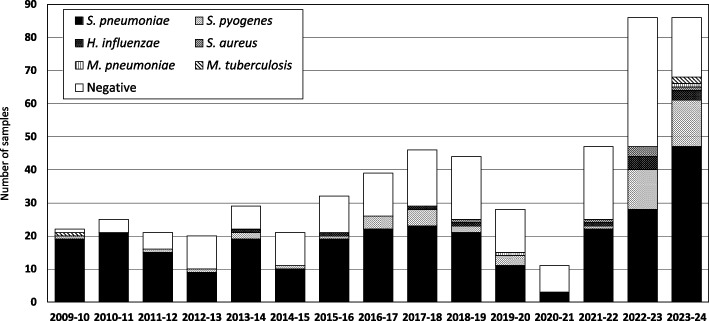
Number of samples from pediatric patients (< 18 years) indicating any identified pathogens (Portugal, 2009/10 – 2023/24)

The distribution of bacterial species identified in our samples is presented in the Fig. 1. Most samples were positive for *S. pneumoniae* (*n* = 289, 53.1%), followed by 48 samples positive for *S. pyogenes* (8.8%). *H. influenzae* and *S. aureus* were found in smaller numbers (*n* = 12, 2.2% and *n* = 6, 1.1% each, respectively), three samples were positive for *M. tuberculosis* (0.6%) and two for *M. pneumoniae* (0.4%). *S. agalactiae* was not detected in any sample. In additional Table 2 we present the distribution into age groups of the pathogens detected in the samples. *S. pyogenes* was more frequent among samples collected in children up to 23-months of age, whereas *S. pneumoniae* was detected more frequently in patients 2–17 years of age (Fisher exact test, *p* < 0.001).

In 13 samples, two bacterial species were detected: *S. pneumoniae* and *S. pyogenes* (*n* = 7), *S. pneumoniae* and *H. influenzae* (*n* = 4), *S. pneumoniae* and *S. aureus* (*n* = 1), and *S. pneumoniae and M. tuberculosis* (*n* = 1).

## Discussion

Despite the high uptake of PCV13 in Portugal, most PCPs in our study were still caused by *S. pneumoniae*. *S. pyogenes* ranked second, with approximately half of the positive cases (26/48) being detected in the two last epidemiological years (Fig. 1), concurrent with an outbreak of pediatric invasive GAS infections in Portugal [6]. However, PCPs due to *S. pyogenes* were also detected in almost all epidemiological years, reinforcing the importance of this pathogen even outside outbreak contexts. In contrast, in a single center in Australia, the increase of *S. pyogenes* in PCP in 2022–2023 was much more marked and contributed significantly to a rise of PCP incidence [7].

Another difference in our study from previous reports is the proportion of infections caused by *S. aureus*, since it is frequently described as a major agent, often reported more frequently than *H. influenzae* [5, 8], in contrast to the situation in Portugal where there were twice as many infections by *H. influenzae* (*n* = 12, 2.2%). As with *S. pyogenes* infections, approximately half of the *H. influenzae* cases were reported in the last two epidemiological years (4 cases in 2022–2023 and 3 cases in 2023–2024). In these two seasons, an increase in the number of submitted samples was seen, after a marked decline in 2020–2021. We do not believe this is due to any change in our surveillance but to be consistent with the resurgence of respiratory diseases in the post-pandemic years noted in several countries [9]. From 2022–2023 to 2023–2024, a decrease in samples in which no bacterial agent was detected and an increase in the proportion of samples positive for *S. pneumoniae* were also found (Fig. 1). Similarly, the single center study from Australia found that the post-pandemic increase in incidence of PCP was accompanied by a decrease of the number of samples in which no pathogen was identified [7]. As in other countries, in Portugal there was a rebound in overall invasive pneumococcal disease in children in 2022–2023 [10], with this trend persisting through at least 2023–2024. The increase in PCP samples seen in 2022–2024 was driven mostly by serotype 3, which was also the dominant serotype pre-COVID-19 pandemic [1, 2, 10].

During the autumn of 2023 there was an increase of *M. pneumoniae* infections in several countries of the northern hemisphere. However, the number of infections reported in two Portuguese hospitals between April 2022 and September 2023 remained low [11] and in our study no increase of PCP cases due to *M. pneumoniae* was seen.

We found cases where the DNA of two bacterial species was detected, suggesting possible co-infections in these cases, as already reported [8]. Since we have no data regarding the immune status or other comorbidities of the patients, we cannot say if these occurred in a particularly susceptible population.

Our work has limitations. Since we did not collect any clinical information, we cannot explore potential differences in severity between cases caused by different species nor if cases where no pathogen was identified correspond to patients under antimicrobial treatment for longer before sample collection. Additionally, although the study involved both pediatric and microbiology departments, it was not designed for the estimation of PCP incidence, which may have changed during the study period.

More than two decades after extensive use of PCVs and after the major perturbation induced by the COVID-19 pandemic, PCPs are still caused mainly by *S. pneumoniae*. Diagnostic tests and empiric therapy of PCPs should continue to focus on *S. pneumoniae*, even in countries with a high PCV uptake. However, the etiology of more than a third of cases in our study remained unknown and other microbial agents can emerge as important causes of infection, reinforcing the need to expand NAATs to identify the etiology of these complicated infections.

## Supplementary Information

Supplementary Material 1.

## Data Availability

Data is provided within the manuscript or supplementary information files.

## Abbreviations

NAAT: Nucleic acid amplification test
PCP: Pediatric complicated pneumonia
PCV: Pneumococcal conjugate vaccine
PCV13: 13-Valent pneumococcal conjugate vaccine
