# Correction to: Pneumococci remain the main cause of complicated pediatric pneumonia in the post-pandemic era despite extensive pneumococcal vaccine use

**DOI:** 10.1186/s41479-025-00179-7

**Published:** 2025-09-15

**Authors:** Joana Gomes‑Silva, Marcos D. Pinho, Ana Friaes, Mario Ramirez, Jose Melo‑Cristino, Catarina Silva‑Costa, the Portuguese Group for the Study of Streptococcal Infections, Margarida Pinto, Miguel Seruca, João Marques, Isabel Peres, Teresa Pina, Isabel Lourenço, Cristina Marcelo, Isabel Daniel, Odete Chantre, Vasco Mendes, Marília Gião, Rui Ferreira, Rui Tomé Ribeiro, Celeste Pontes, Luísa Boaventura, Teresa Reis, Henrique Oliveira, Catarina Chaves, Mariana Silva, Ana Aguiar, Hugo Loureiro, Adriana Pedrosa, Hermínia Costa, Maria Fátima Silva, Maria Amélia Afonso, Mariana Fardilha, Natália Novais, Isabel Brito, Luís Marques Lito, Ana Bruschy Fonseca, Maria Ana Pessanha, Elsa Gonçalves, Teresa Morais, Cristina Toscano, Elisabete Cristovam, Paulo Lopes, Angelina Lameirão, Gabriela Abreu, Aurélia Selaru, Ana Paula Mota Vieira, Margarida Tomaz, Cláudia Ferreira, Marta Nicolau, Ana Paula Castro, Virgínia Lopes, Hugo Cruz, Fernando Fonseca, Nádia Martins, Carla Leite, Ana Paula Castro, Filipa Vicente, Margarida Pereira, Ilse Fontes, Maria Paula Falcão, Rui Semedo, Gina Marrão, Filipa Silva, Manuela Ribeiro, Helena Gonçalves, Alberta Faustino, Maria Cármen Iglesias, Adriana Coutinho, Ana Bela Correia, Luísa Gonçalves, Elzara Aliyeva, Sandra Schäfer, Clara Portugal, Isabel Monge, José Diogo, Filipa Fortunato, Leonardo Carneiro, José Marta, Nadiya Kruptsala, Cláudia Fidalgo, Raquel Diaz, Sónia Ferreira, Inês Cravo Roxo, Isabel Vale, Maria João Tomás, Maria Antónia Read, Valquíria Alves, Margarida Monteiro, João Faria, José Mota Freitas, Sandra Vieira, Elsa Calado, Bruno Miguel, L Nogueira Martins, Maria Favila Menezes, Maria José Rego de Sousa, Maria Calle, Mariana Bettencourt Viana, Marvin Oliveira, Hugo Macedo, Vitória Rodrigues, Sofia Marques, Joana Selada, Patrícia Pereira, Manuela Azevedo, Jesuína Duarte, Joana Bernardo, Inês Tapadinhas, Ana Filipa Resende, Andreia Bernardo, Luísa Oliveira, Susana Banza, Ezequiel Moreira, Carla Ferreira, Adília Vicente, Cristina Bragança, Maria Lucas, Paula Gouveia Pestana, Patrícia Amantegui, Cristina Mota Preto, Sara F. Sampaio, Ana Jesus, Marisol Lourinha, the Portuguese Study Group of Invasive Pneumococcal Disease of the Pediatric Infectious Disease Society, Catarina Gouveia, Teresa Tomé, Mónica Rebelo, Ana Teixeira, Maria João Virtuoso, Nancy Guerreiro, Fernanda Rodrigues, Cristina Resende, Sónia Aires, Agostinho Fenandes, Filipa Prata, Marisa Vieira, Rita Morais, Diana Moreira, Isabel Carvalho, Alexandra Costa, Ana Teixeira, Cristina Ferreira, Graça Seves, Laura Marques, Ana Braga, Margarida Guedes, Maria José Dinis, Eurico Gaspar, Bernardo Camacho, Céu Novais, Maria Manuel Zarcos, Margarida Tavares, Manuela Costa Alves, Sofia Lima, Carla Cruz, Manuela Brandão, Paula Correia, Sofia Fraga, João Franco, Sílvia Almeida, Cristina Faria, Sofia Arosa, Florbela Cunha, Hugo Rodrigues, Joaquim Cunha, Cláudia Monteiro, Estela Veiga, Fernanda Pereira, Manuela Ferreira, Álvaro Sousa, Francisca Lopes, Sara Santos, Ana Luísa Teixeira, Fernanda Marcelo, Pedro Carvalho, Filomena Pereira, Gustavo Rodrigues, Marta Cabral, Maria Ana S. Nunes, Pedro Flores, Manuel Cunha, Dora Gomes, João Calado Nunes, Rosário Massa, Fátima Nunes, Isabel Monteiro, Cristina Didelet, António Salgado, Luís Gonçalves

**Affiliations:** 1 Instituto de Medicina Molecular, Faculdade de Medicina, Universidade de Lisboa, Av. Prof. Egas Moniz, Lisbon, PT 1649-028 Portugal; 2 Unidade Local de Saúde de São José, Lisboa, Portugal; 3 Unidade Local de Saúde Algarve, Faro e Portimão, Portugal; 4 Unidade Local de Saúde Coimbra, Coimbra, Portugal; 5 Unidade Local de Saúde Entre Douro e Vouga, Sta Maria da Feira, Portugal; 6 Unidade Local de Saúde Baixo Mondego, Figueira da Foz, Portugal; 7 Unidade Local de Saúde Santa Maria, Lisboa, Portugal; 8 Unidade Local de Saúde Lisboa Ocidental, Lisboa, Portugal; 9 Unidade Local de Saúde Gaia/Espinho, Vila Nova de Gaia e Espinho, Portugal; 10 Unidade Local de Saúde Alto Ave, Guimarães, Portugal; 11 Unidade Local de Saúde Baixo Alentejo, Beja, Portugal; 12 Unidade Local de Saúde Santo António, Porto, Portugal; 13 Unidade Local de Saúde Póvoa do Varzim/Vila do Conde, Póvoa do Varzim e Vila do Conde, Portugal; 14 Unidade Local de Saúde Trás-os-Montes e Alto Douro, Vila Real, Peso da Régua e Chaves, Portugal; 15 Serviço de Saúde da Região Autónoma da Madeira, Funchal, Portugal; 16 Unidade Local de Saúde Alto Alentejo, Elvas e Portalegre, Portugal; 17 Unidade Local de Saúde Região de Leiria, Leiria, Portugal; 18 Unidade Local de Saúde de São João, Porto, Portugal; 19 Unidade Local de Saúde Braga, Braga, Portugal; 20 Unidade Local de Saúde Alentejo Central, Évora, Portugal; 21 Hospital dos SAMS, Lisboa, Portugal; 22 Unidade Local de Saúde Amadora/Sintra, Amadora e Sintra, Portugal; 23 Unidade Local de Saúde Almada/Seixal, Almada e Seixal, Portugal; 24 Unidade Local de Saúde Região de Aveiro, Aveiro, Portugal; 25 Unidade Local de Saúde Viseu, Dão - Lafões, Tondela e Viseu, Portugal; 26 Unidade Local de Saúde Matosinhos, Matosinhos, Portugal; 27 Unidade Local de Saúde Estuário do Tejo, Vila Franca de Xira, Portugal; 28 Unidade Local de Saúde Alto Minho, Ponte de Lima e Viana do Castelo, Portugal; 29 Centro de Medicina Laboratorial Germano de Sousa, Lisboa, Portugal; 30 Unidade Local de Saúde Tâmega e Sousa, Amarante e Guilhufe, Portugal; 31 Laboratório Synlab, Lisboa, Portugal; 32 Unidade Local de Saúde Arrábida, Setúbal, Portugal; 33 Unidade Local de Saúde Médio Ave, Santo Tirso e Vila Nova de Famalicão, Portugal; 34 Unidade Local de Saúde Oeste, Caldas da Rainha, Portugal; 35 Unidade Local de Saúde Cova da Beira, Covilhã, Portugal; 36 Centro de Medicina Laboratorial Germano de Sousa Açores, Ponta Delgada, Portugal; 37 Unidade Local de Saúde do Arco Ribeirinho, Barreiro e Montijo, Portugal; 38 Hospital Particular do Algarve, Faro, Portugal; 39 Unidade Local de Saúde Loures/Odivelas, Loures, Portugal; 40 Unidade Local de Saúde do Nordeste, Bragança, Portugal; 41 Unidade Local de Saúde Castelo Branco, Castelo Branco, Portugal; 42 Unidade Local de Saúde Guarda, Guarda, Portugal; 43 Instituto Português de Oncologia, Lisboa, Portugal; 44 Hospital Lusíadas, Lisboa, Portugal; 45 Hospital da Luz, Lisboa, Portugal; 46 Hospital da Cruz Vermelha, Lisboa, Portugal; 47 Hospital CUF Descobertas, Lisboa, Portugal; 48 Hospital de Cascais, Cascais, Portugal; 49 Hospital da Horta, Horta, Portugal; 50 Unidade Local de Saúde da Lezíria, Santarém, Portugal; 51 Unidade Local de Saúde do Médio Tejo, Abrantes, Tomar e Torres Novas, Portugal; 52 Hospital do Santo Espírito, Angra do Heroísmo, Portugal; 53 Hospital do Divino Espírito Santo, Ponta Delgada, Portugal

**Correction: Pneumonia 16, 26 (2024)**

**https://doi.org/10.1186/s41479-024-00151-x**

Following publication of the original article [1], the authors identified an error in Fig. 1. The legend in the figure no longer included the formatting as in the accepted manuscript. The correct figure is given below.

The incorrect Figure 1:


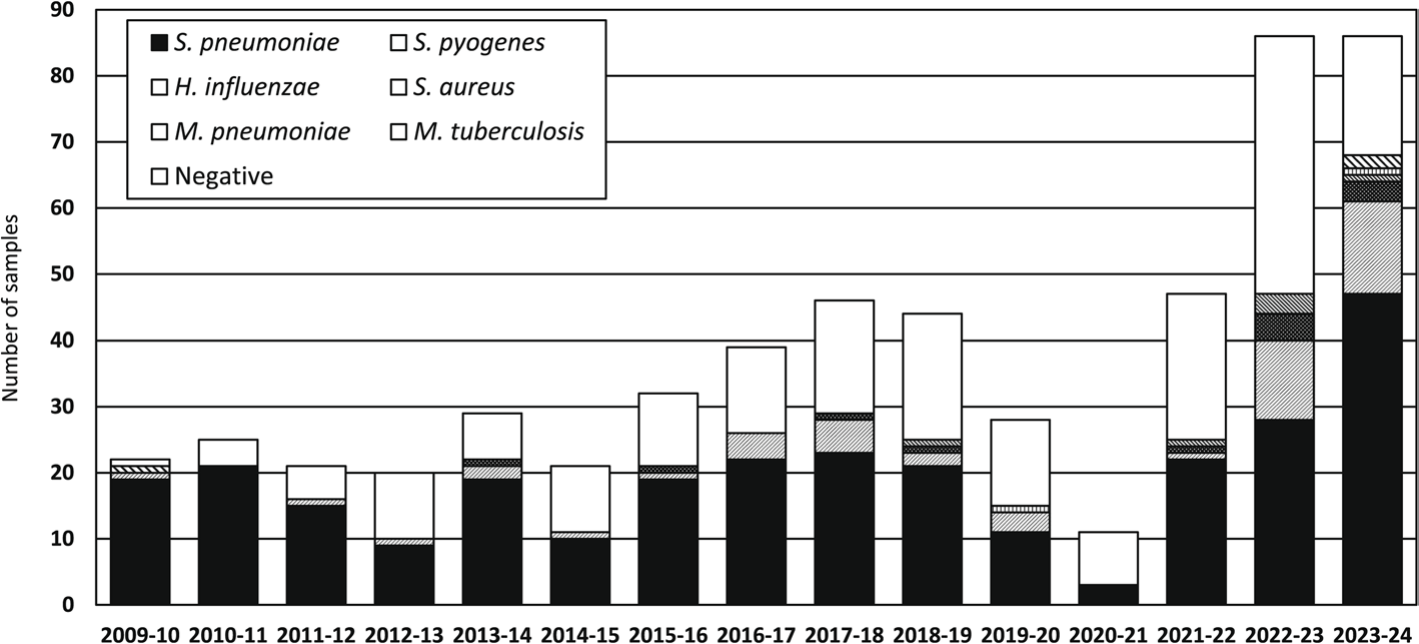


The correct Figure 1:

**Fig. 1:**
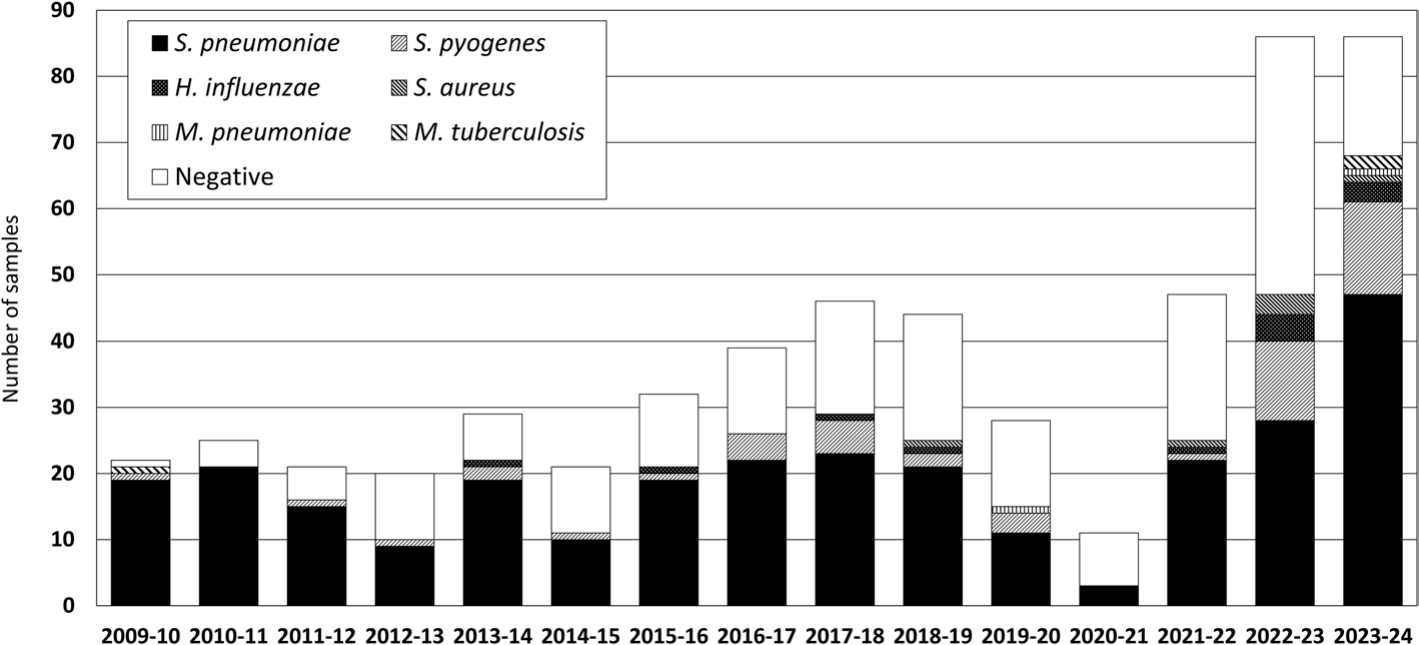
has been updated above and the original article [1] has been corrected
