# Supplementary material for: Pneumococci remain the main cause of complicated pediatric pneumonia in the post-pandemic era despite extensive pneumococcal vaccine use
Source: Pneumonia (Nathan). 2024 Nov 25;16:26. doi: 10.1186/s41479-024-00151-x (PMC11587768; doi:10.1186/s41479-024-00151-x)
Supplement: Supplementary file 1 — Supplementary Material 1. [file 41479_2024_151_MOESM1_ESM.pdf]

Table S1: Number of requests for PCR testing and confirmed positive samples for at least one of the bacterial species included in the PCR schema, in pediatric patients in Portugal, 2010-2024

Samples	Epidemiological Year[1]															Total
	2009-10	2010-11	2011-12	2012-13	2013-14	2014-15	2015-16	2016-17	2017-18	2018-19	2019-20	2020-21	2021-22	2022-23	2023-24	
Positive	20	21	15	10	21	11	21	26	29	24	15	3	25	45	61	347
Negative	1	4	5	10	7	10	11	13	17	19	13	8	22	39	18	197
Total Requests	21	25	20	20	28	21	32	39	46	43	28	11	47	84	79	544

[1]Epidemiological years were defined as spanning from week 26 to week 25 of the following year and the study included samples recovered between July 2010 (week 26) and June 2024 (week 25).

Table S2: Number of samples in which each tested pathogen was identified in the different age groups in Portugal, 2010-2024

Pathogen[1]	Age group (number of positive samples)[2]		
	0-23 months	2-5 years	6-17 years
H. influenzae	6	4	2
M. pneumoniae	0	1	1
M. tuberculosis	0	0	3
S. aureus	2	2	2
S. pyogenes	19	21	8
S. pneumoniae	40	172	74

[1]In 13 samples two pathogens were detected (see text for details)
[2]For three samples, patient age information was not available
